# Predicting outcome of patients with prolonged disorders of consciousness using machine learning models based on medical complexity

**DOI:** 10.1038/s41598-022-17561-w

**Published:** 2022-08-05

**Authors:** Piergiuseppe Liuzzi, Alfonso Magliacano, Francesco De Bellis, Andrea Mannini, Anna Estraneo

**Affiliations:** 1 IRCCS Fondazione Don Carlo Gnocchi ONLUS, Via di Scandicci 269, Florence, Italy; 2 Scuola Superiore Sant’Anna, Istituto di BioRobotica, Viale Rinaldo Piaggio 34, Pontedera, Italy; 3 Fondazione Don Carlo Gnocchi ONLUS, Scientific Institute for Research and Health Care, Via Quadrivio, Sant’Angelo dei Lombardi, Italy; 4 Unità di Neurologia, Santa Maria della Pietà General Hospital, Via della Repubblica 7, Nola, Italy

**Keywords:** Disorders of consciousness, Biomedical engineering, Mathematics and computing

## Abstract

Patients with severe acquired brain injury and prolonged disorders of consciousness (pDoC) are characterized by high clinical complexity and high risk to develop medical complications. The present multi-center longitudinal study aimed at investigating the impact of medical complications on the prediction of clinical outcome by means of machine learning models. Patients with pDoC were consecutively enrolled at admission in 23 intensive neurorehabilitation units (IRU) and followed-up at 6 months from onset via the Glasgow Outcome Scale—Extended (GOSE). Demographic and clinical data at study entry and medical complications developed within 3 months from admission were collected. Machine learning models were developed, targeting neurological outcomes at 6 months from brain injury using data collected at admission. Then, after concatenating predictions of such models to the medical complications collected within 3 months, a cascade model was developed. One hundred seventy six patients with pDoC (M: 123, median age 60.2 years) were included in the analysis. At admission, the best performing solution (k-Nearest Neighbors regression, KNN) resulted in a median validation error of 0.59 points [IQR 0.14] and a classification accuracy of dichotomized GOS-E of 88.6%. Coherently, at 3 months, the best model resulted in a median validation error of 0.49 points [IQR 0.11] and a classification accuracy of 92.6%. Interpreting the admission KNN showed how the negative effect of older age is strengthened when patients’ communication levels are high and ameliorated when no communication is present. The model trained at 3 months showed appropriate adaptation of the admission prediction according to the severity of the developed medical complexity in the first 3 months. In this work, we developed and cross-validated an interpretable decision support tool capable of distinguishing patients which will reach sufficient independence levels at 6 months (GOS-E > 4). Furthermore, we provide an updated prediction at 3 months, keeping in consideration the rehabilitative path and the risen medical complexity.

## Introduction

After a severe acquired brain injury (sABI), patients can exhibit prolonged (> 28 days from onset) disorders of consciousness (pDoC^1,2^). In particular, eye-opening (spontaneously or in response to stimuli) in absence of any intentional behaviors defines the Vegetative State/Unresponsive Wakefulness Syndrome (VS/UWS^3^), whereas presence of inconsistent but reproducible intentional behaviors is a clinical marker of minimally conscious state (MCS^1,4^). The mortality rate of patients with pDoC is higher in the first than in the second year after brain injury in both diagnostic groups^5^, and consciousness recovery occurs more frequently in patients in MCS than in patients in VS/UWS^6–8^. To date, the prognostication of patients with pDoC admitted to neurorehabilitation setting (i.e. in the post-acute phase) is based on markers strictly related to brain damage and patients’ characteristics at admission. Previous studies identified demographic/etiology information (i.e., younger age, female sex, traumatic etiology, time since injury^6,9–12^), clinical characteristics (i.e., the level of responsiveness assessed by validated behavioral scales^13^) and neurophysiological findings (i.e., presence of bilateral somatosensory evoked potentials^14^) as predictors of a better recovery in the medium-long term, both in terms of survival and functional recovery. However, the high clinical complexity and instability of patients with pDoC requires to take into account further factors that could not be present at admission^1,3^. Among these factors, the occurrence of severe medical complications (MCs) during the hospital stay seems to have a large impact on outcome up to one year and longer^15^. Most MCs in pDoC are directly related to the brain injury, as paroxysmal sympathetic hyperactivity^16^ or epileptic seizures^17^, or are developed as a consequence of severe disability or medical devices, e.g. heterotopic ossifications^18^ and pneumonia^19^. The occurrence of such MCs is associated with a higher frequency of rehospitalization and with a worse functional outcome in acute^20,21^ and rehabilitative settings^15,17,22–24^ respectively.

Given the clinical variability of patients with pDoC during the rehabilitative path, Clinical Decision Support Tools can play a role in supporting the clinical team. In general terms, it is a technical solution devoted to improving healthcare delivery by enhancing medical decisions with available knowledge. Such knowledge may result from machine learning (ML)-based methods, trained on patients’ clinical and instrumental data^25^. In particular, learning algorithms can integrate patient information from many, interacting, sources and extract from data their relations with a prognostic outcome. Advantages of support tools in healthcare include the possibility to contain costs, improve the clinical workflow, increase patients’ safety, support diagnosis, and promote treatment personalization^25,26^. In this regard, concerning pDoC patients, ML-enabled solutions were proposed, targeting prognostic estimations for decannulation^27^ and recovery of consciousness^28–31^. To our knowledge, previous solutions adopted data recorded at early stage after admission, disregarding the occurrence of MCs within the rehabilitative path. Given the high relevance of MCs^15,18,32^, we are convinced that intaking data collected during the hospital stay would foster the improvement of prognostic predictions. Up to our knowledge, the information on complications has always been merged together with clinical and instrumental variables, without evaluating whether complications could improve the prediction made at admission and/or the mutual influence between arisen complications and admission prognosis.

Here, we used a ML approach to a retrospective analysis of data from a multicenter longitudinal study on a cohort of patients with pDoC. First, we aimed at predicting clinical outcome at 6 months post-injury based on demographical, etiology and clinical data collected at admission in intensive rehabilitation units. Thereafter, the prediction model had been adjusted with information on MCs collected within the first 3 months of the rehabilitative path. In this context, such innovative use of ML models allowed to create algorithms which move from a cross-sectional-based prediction of outcomes, in favor of a dynamic prediction system that can be updated during the patient stay^33^. Among the ML techniques chosen, simple algorithms were employed in order to maximize generalization capabilities and to understand whether simple, classical models were already sufficient to predict the outcome. Lastly, to bolster interpretability of the results, explainability methods based on Shapley Values were applied to the best performing ML solutions in the form of the SHapley Additive exPlanations (SHAP) technique^34,35^.

## Materials and methods

### Study design and population

The present study retrospectively analyzed data on a large cohort of patients with pDoC enrolled in a multi-center, observational, longitudinal design (see details in Estraneo et al.^36^). Inclusion criteria were: (i) age ≥ 18 years; (ii) diagnosis of pDoC (VS/UWS or MCS) according to standardized criteria for VS/UWS and MCS^4,37^; (iii) traumatic or non-traumatic (i.e., anoxic or vascular) brain injury; (iv) time post-injury ranging from 28 days to 3 months. Exclusion criteria were: (i) mixed etiology; (ii) previous history of neurologic or psychiatric diseases.

### Data collection and outcome definition

**Figure 1:**
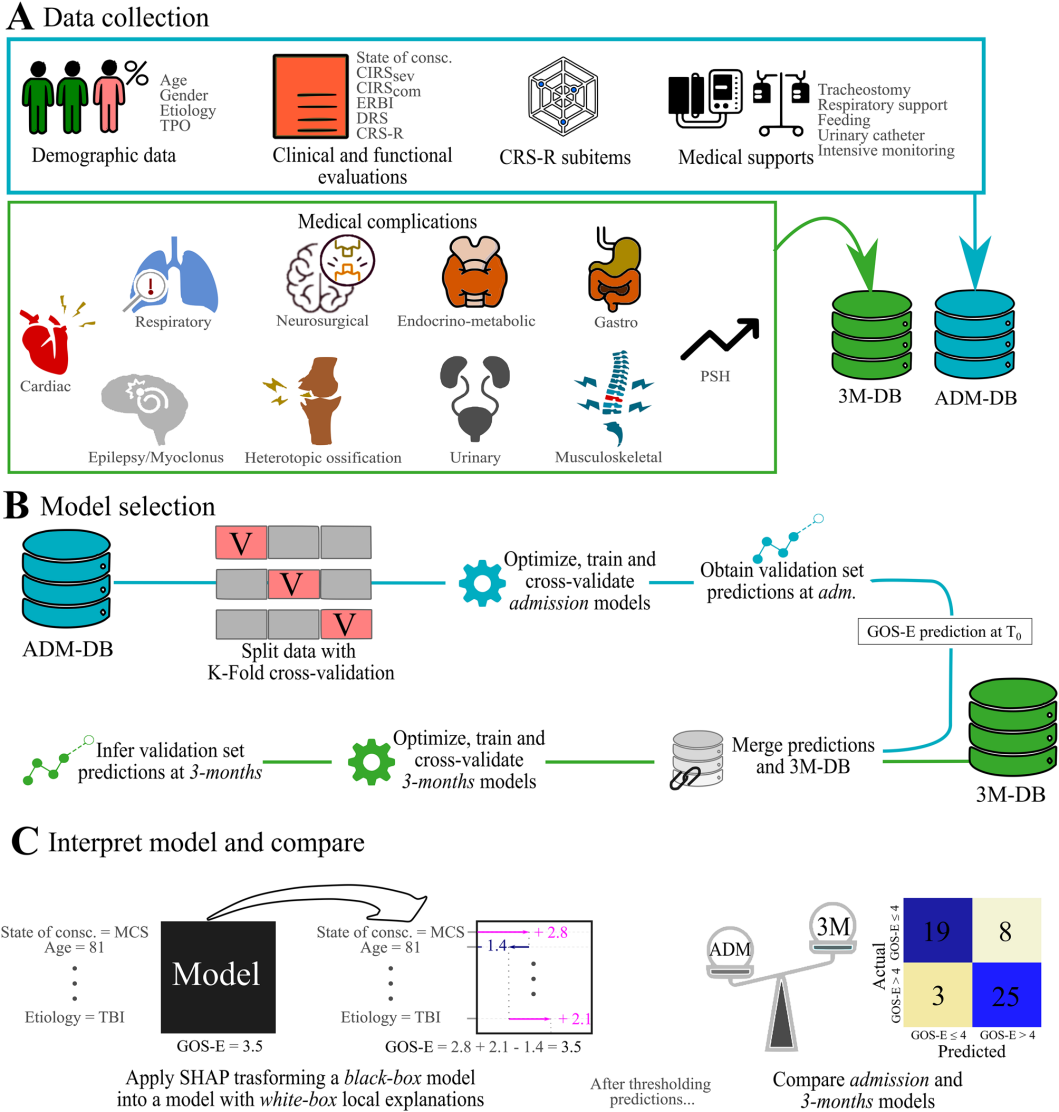
Work pipeline starting from data collection (**A**) at the admission (light blue, ADM-DB) and MCs at 3 months (green, 3M-DB). In the model selection, the k-fold cross-validated admission model predictions are attached, following the same k-fold split, to the MCs at 3 months (**B**). Together, these data are used to train and cross-validate the 3-month model. Both models have the GOS-E value at discharge as target. (**C**) Representative example of how weights assigned patient-wise to the independent variables contribute to the overall prediction (left) and how predictions are dichotomized for model comparison (right).

Data collected at study entry included demographics (age, sex), medical history (injury timing, etiology), the best total and sub-scores out of at least five^38,39^ Coma Recovery Scale-Revised (CRS-R^40^) evaluations, the level of functional disability indexed by the Disability Rating Scale total score (DRS^41,42^), the level of clinical complexity as measured by the Early Rehabilitation Barthel Index (ERBI^43^), medical comorbidities before the brain injury as assessed by the Cumulative Illness Rating Scale (CIRS^44^), presence of medical devices (e.g. for supporting respiratory functions, feeding). Full details about variable collection at admission are reported in Estraneo et al.^36^ and in Fig. 1A.

Moreover, MCs occurring in the first 3 months of neuro-rehabilitation stay were assessed by direct clinical observation of hospital staff and grouped into 10 categories, and their severity was rated on a 1–3 scale (mild, moderate or severe) on the basis of the ‘intensity’ of the required therapeutic interventions according to Estraneo et al.^15^. A MCs total score (MC_tot_), ranging 0–28, was computed by summing up the ratings in the individual MCs categories. The checklist for MCs categorization is described in Supplementary Materials A.

The primary outcome was the clinical diagnosis and functional state at 6 months post-injury, as assessed by the Glasgow Outcome Scale-Extended (GOS-E^45^). For the purpose of statistical analysis, the GOS-E score was dichotomized into GOS-E > 4 indicating ‘favorable outcome’ (i.e., from low-moderate disability to good recovery) vs. GOS-E ≤ 4 indicating ‘unfavorable outcome’ (i.e., from high-severe disability to death). See Supplementary Materials B for GOS-E description.

### Statistical analysis

Continuous variables were expressed via medians and interquartile ranges, whereas categorical variables as counts and percentages. Univariate correlations were computed between independent variables and the target set to GOS-E > 4 versus GOS-E ≤ 4. Specifically, logistic regressions were applied for continuous independent variables and chi-square test for categorical independent variables. When appropriate, Fisher’s exact test was adopted. The level of significance was set at p-value = 0.05 (2-tailed).

### Model selection

Data at admission were split using a k-fold cross-validation strategy with the number of folds set to five. By such strategy, three models were compared: Elastic-Net (EN), Orthogonal-Matching Pursuit (OMP), K-Nearest-Neighbor (KNN) and a Support Vector Regressor (SVR) (Fig. 1B). The optimization of models hyperparameters aimed to minimize k-fold cross-validation error with the target set equal to the GOS-E value at 6 months. Each of the training set in cross-validation splits was resampled to overcome dataset imbalance, using the Synthetic Minority Oversampling TEchnique (SMOTE)^46^. Predictions from models trained using data at admission were attached to the 3-months MC dataset, split using the k-fold cross-validation indexes adopted in the previous step. For each of the admission models’ predictions, three 3-month models were deployed, specifically an EN, an OMP and a KNN. Consequently, nine models resulted using the full dataset, considering all 3 × 3 combinations of regressors. K-fold cross-validation accuracies were computed for all models and compared. Furthermore, actual and predicted values were dichotomized in GOS-E > 4/GOS-E ≤ 4 in order to retrieve accuracy, sensitivity and specificity of the dichotomized outcome classification.

Optimization was performed using the Optuna library^47^ and all ML models were implemented utilizing the Scikit-Learn library^48^.

### Model interpretability

Different methods to interpret Machine Learning black-boxes are currently available in literature^49^. Such methods allow to investigate the feature contribution to the predictions. Elastic-Net or OMP, and more in general Generalized Linear Models, already allow for interpretability and explainability measures, by assigning to each independent variable the height of its related regression coefficient ${\upbeta }_{i}$ and therefore calculating the effect of the feature vector $x$ onto the prediction via the product $\upbeta x$. Nevertheless, given the k-fold cross-validation implementation, each patient ends up $k-1$ times in a training set and only once in a validation set. Consequently, k models will result in a parameter estimate ${\upbeta }_{N}$. Accordingly, evaluating feature importance via averaging the $k$ coefficients β is possible, but has two major drawbacks. Firstly, the resulting variability in the parameters estimates can be relevant. Secondly, Shapley values does not only provide mean trends derived from the full data as for β regression coefficients but offers a patient-wise estimation of feature contribution to the predictions (Fig. 1C).

### Ethics

This study was approved by the Ethics Committee of the coordinator center (Fondazione Pascale IRCCS, Napoli, Protocol number 1/16, 15.06.2016) and confirmed by the local ethics committees of each center involved in the study and performed according to the ethical standards of the Declaration of Helsinki (1964) and its later amendments. We have to specify that the Ethics Committee of Fondazione Pascale is the Campania Regional Reference Center for the Scientific Institutes of Research and Care (IRCCS). This is the reason why the Ethics Committee of Fondazione Pascale was in charge of approving the project, even though no author is affiliated there. The list of 23 local ethics committees/institutional review boards which participated in the study is reported in the Supplementary Materials C. The Legally Authorized Representative of all patients enrolled in the study provided written informed consent. The original forms were collected and stored at each participant centre in accordance with national regulation on the protection of personal data, and anonymized data were then centralized in one secured database.

## Results

### Univariate analysis

Overall, 176 patients with DoC were included in the study (104 males; median age = 60.2 years [IQR 21.7]; median time post-insult = 1.3 months [IQR 1.23]) of whom the 51.7% (n = 91) were in a VS/UWS (Table 1). Detailed data at study entry were reported elsewhere^36^.

**Table 1:** Admission descriptive and inferential statistics for admission variables.

	GOS-E ≤ 4	GOS-E > 4	OR/[χ2]/{Fisher's}	OR, 95% CI	p-value
**Clinical and functional evaluations**					
**Age, years**	63.43 [21.82]	51.23 [36.04]	0.960	0.941–0.980	< 0.001
Gender, M	104 (70.3)	19 (67.9)	[0.065]	–	0.799
Time post-insult, days	1.38 [1.20]	1.08 [1.19]	0.685	0.390–1.202	0.188
CIRS_sev_	0.36 [0.57]	0.36 [1.05]	1.410	0.677–2.937	0.359
CIRS_com_	1.5 [3]	1 [5]	1.048	0.894–1.228	0.565
**Clinical State, MCS**	63 (42.6)	22 (78.6)	[12.223]	–	0.001
**Etiology**			[3.431]	–	0.330
*TBI*	50 (33.8)	11 (39.3)	[0.315]	–	0.575
*Anoxic*	29 (19.6)	3 (10.7)	{1.248}	–	0.422
*Vascular*	60 (40.5)	14 (50.0)	[0.865]	–	0.352
ERBI	− 175 [50]	− 225 [81]	0.994	0.986–1.003	0.212
**DRS**	24 [4]	21 [7]	0.735	0.641–0.842	< 0.001
Pressure Sores	63 (42.6)	7 (25.0)	[3.034]	–	0.082
Lacunar Skull	21 (14.2)	5 (17.9)	[0.252]	–	0.616
**CRS-R total score**	7 [6]	12.5 [6]	0.752	0.669–0.846	< 0.001
Auditory	1 [1]	2.5 [2]	2.926	1.853–4.620	< 0.001
Visual	1 [2]	3 [2]	1.865	1.346–2.585	< 0.001
Motor	2 [2]	3 [3]	1.962	1.434–2.683	< 0.001
Oro-motor	1 [0]	2 [1]	3.948	2.023–7.690	< 0.001
Communication	0 [0]	1 [1]	5.008	2.200–11.399	< 0.001
Arousal	2 [1]	2 [0]	3.384	1.794–8.201	0.001
**Supports**					
Resp. Support			[0.894*]	–	0.744
*Autonomous*	71 (48)	16 (57.1)	[0.792]	–	0.373
*Autonom. w. O2*	68 (45.9)	11 (39.3)	[0.422]	–	0.516
*Ventilation*	9 (6.1)	2 (3.6)	[0.277]	–	0.706
Tracheostomy	141 (95.3)	24 (85.7)	{3.669}	–	0.055
**Feeding support**			[14.987]	–	0.004
*PEG*	92(62.2)	10 (35.7)	[6.759]	–	0.009
*NGT*	50 (33.8)	14 (50)	[2.676]	–	0.102
*Per OS*	5 (3.4)	1 (3.6)	[0.003]	–	0.959
Urinary catheter	146 (98.6)	28 (100)	{0.383}	–	1.000
Intensive monitoring	80 (54.1)	20 (71.4)	[2.897]	–	0.100

*CIRS:* Cumulative Illness Rating Scale, *MCS* Minimally Conscious State, *TBI* Traumatic Brain Injury, *ERBI* Early Rehabilitation Barthel Index, *DRS* Disability Rating Scale, *CRS-R* Coma Recovery Scale-Revised, *PEG* Percutaneous endoscopic gastrostomy, *NGT* nasogastric tube.

Younger age, entry diagnosis of MCS (χ^2^ = 12.22, p = 0.001), and lower DRS score (OR 0.735; CI 0.641–0.842; p < 0.001) were significantly associated with a more favorable outcome at the 6-month follow-up. Furthermore, the CRS-R total score (OR 0.752; CI 0.669–0.846; p < 0.001) was significantly higher in patients with favorable outcome (median 12.5 points [IQR 6]) than in patients with unfavorable outcome (median 7 [IQR 6]). Similarly, all the CRS-R sub-scores were significantly higher in patients with favorable outcome (p < 0.001). As regard medical devices, the presence of feeding supports was significantly associated with the outcome (χ^2^ = 14.99, p = 0.004). In particular, the presence of percutaneous endoscopic gastrostomy at admission was significantly associated to a worse functional outcome (χ^2^ = 6.759, p < 0.01).

Moreover, patients with a favorable outcome showed a lower MC_tot_ score (OR 0.882; CI 0.793–0.980; p = 0.020). Among the MCs categories, the presence of respiratory complications during the hospital stay was found to be associated with the unfavorable outcome (OR 0.682; CI 0.477–0.974, p = 0.035) (Table 2). No other significant relations were found between the 6-month outcome and single MCs categories (all p > 0.05).

**Table 2:** Admission descriptive and inferential statistics for 3-months complications.

	GOS-E ≤ 4	GOS-E > 4	OR	OR, 95% CI	p-value
**Medical complications**					
Endocrino-metabolic	0 [1]	0 [1]	0.647	0.392–1.069	0.089
Cardiac	0 [1]	0 [1]	0.695	0.447–1.082	0.107
Musculoskeletal	1.5 [3]	1 [2]	0.833	0.623–1.114	0.217
Gastro	0 [1]	0 [1]	0.928	0.617–1.396	0.721
Urinary	0 [1]	0 [1]	0.679	0.438–1.053	0.084
Respiratory	1 [2]	0 [1]	0.682	0.477–0.974	0.035
Neurosurgical	0 [0]	0 [0]	0.929	0.597–1.446	0.745
Epilepsy	0 [0]	0 [0]	0.858	0.448–1.642	0.644
Heterotopic Ossification	0 [0]	0 [0]	1.370	0.820–2.287	0.229
Paroxysmal sympathetic hyperactivity	0 [1]	0 [0]	0.641	0.271–1.513	0.310
Medical Complications_tot_	5 [5]	4 [5]	0.881	0.793–0.980	0.020

### ML model predictions and interpretation

**Figure 2:**
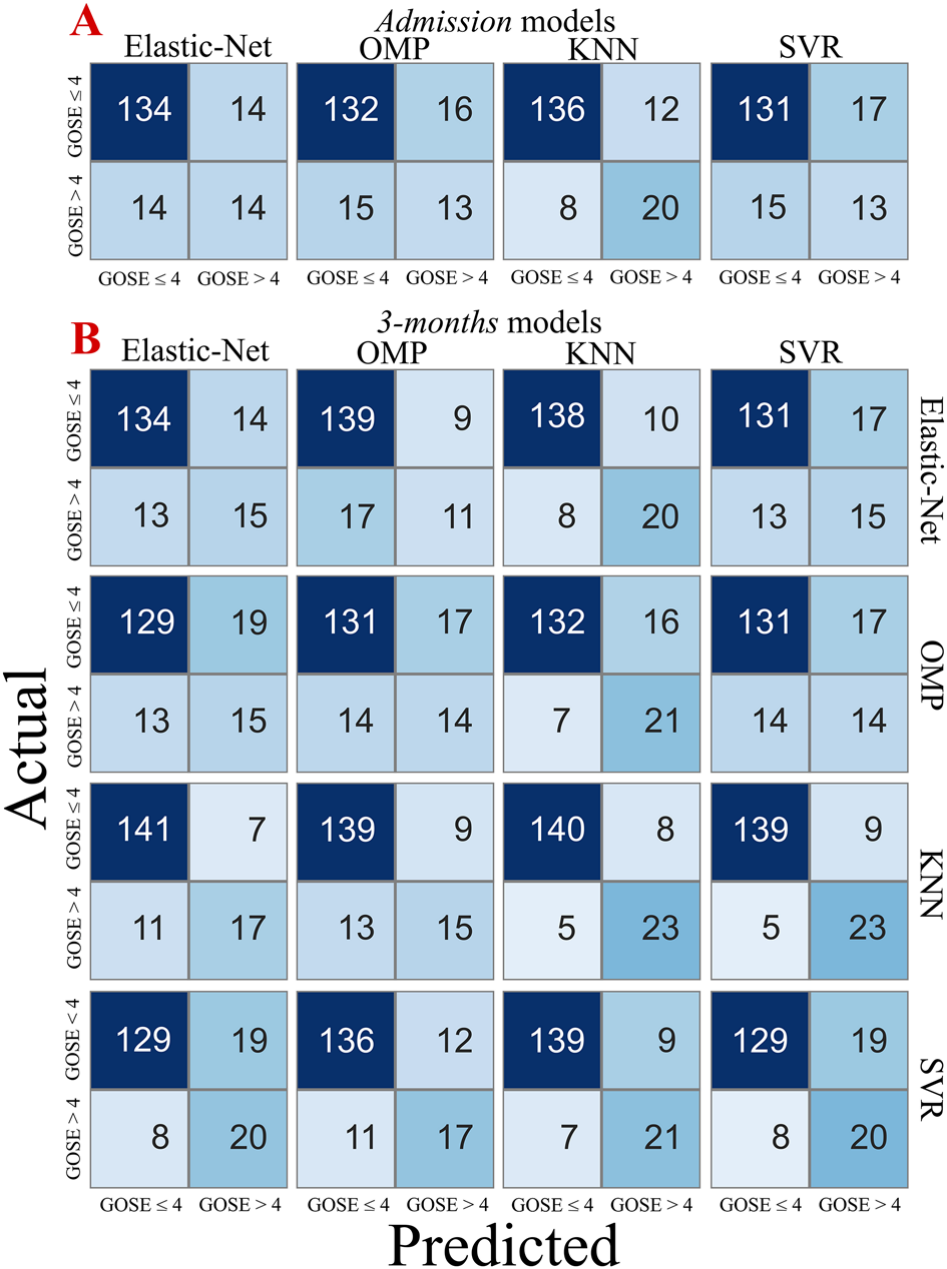
Confusion matrices of classification: predicted values were reported on the x-axis while actual values on the y-axis. In the upper part  (**A**), the confusiom matrices of the four admission models were reported. In the lower part results after the 3-months adaptation of each approach were compared (**B**). All 4 × 4 possible combinations of classifiers were compared: for each of the columns, models built using the prediction of admission model on top were reported.

Optimization results for each of the admission and 3-month models were reported in Supplementary Materials D. Respectively, admission models resulted in a median absolute validation error of 0.70 points [IQR 0.19] for the EN, of 0.71 [IQR 0.25] for the OMP, of 0.63 [IQR 0.14] for the KNN and of 0.73 points [IQR 0.16] for the SVR. By dichotomizing the predictions in GOS-E ≤ 4 and GOS-E > 4, the best solution at admission resulted in the KNN with a classification accuracy of 88.6% (Fig. 2A). The model resulted in a sensitivity of 94.4% and specificity of 62.5%. Among the models built at admission, EN, KNN and SVR improved when concatenating a KNN. Respectively, the EN–KNN combination resulted in a median absolute validation error of 0.53 points [IQR 0.22], the KNN–KNN combination resulted in a median absolute validation error of 0.49 points [IQR 0.20] and the SVR–KNN combination in a median absolute validation error of 0.50 points [IQR 0.23]. The KNN–KNN model, resulted the best performing 3-months solution with a sensitivity of 96.5%, a specificity of 74.2% and classification accuracy of 92.6% (Fig. 2B) very similar to the SVR–KNN combination (sensitivity of 96.5%, specificity of 71.9% and accuracy of 92.0%).

**Figure 3:**
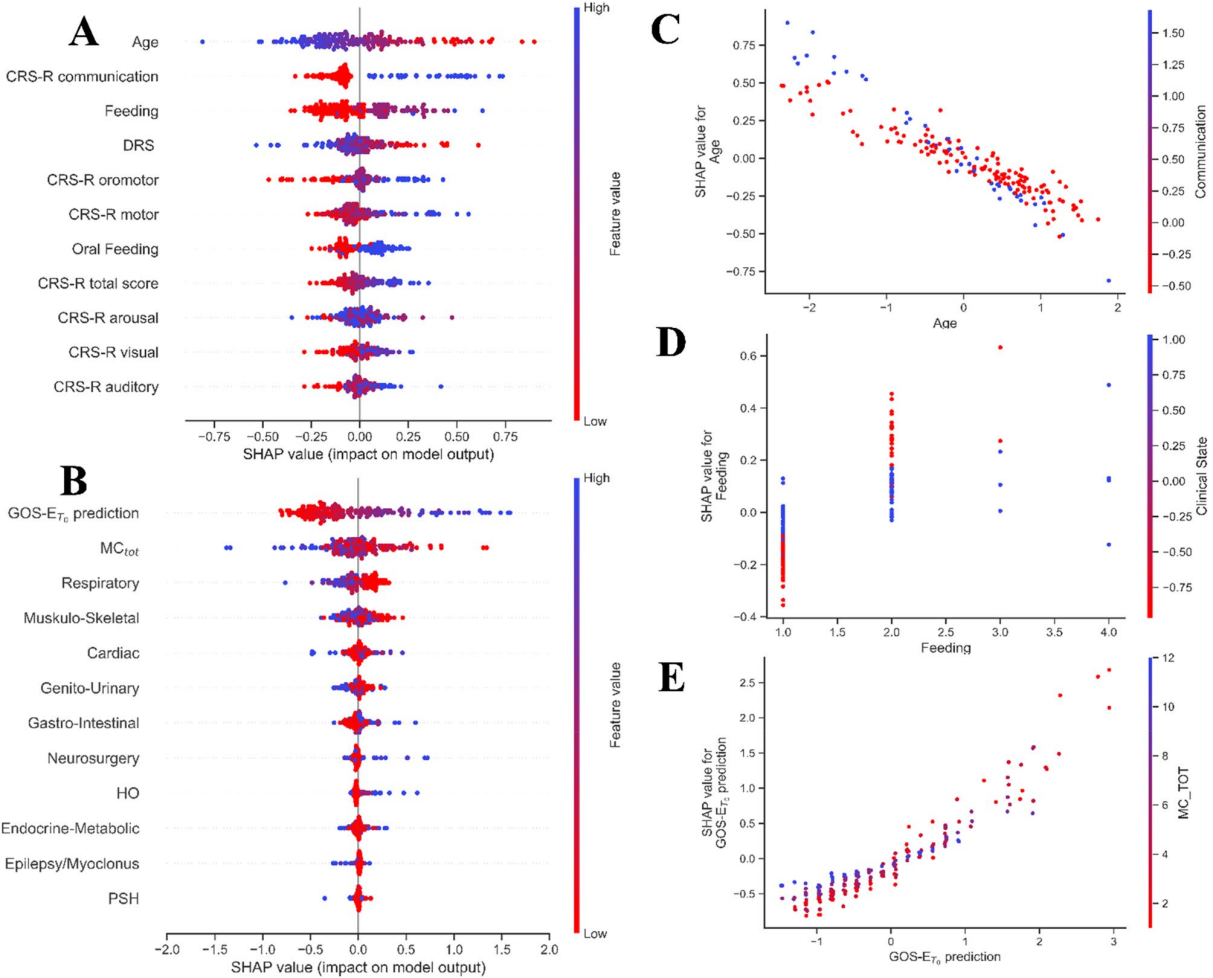
SHAP values were computed for both the admission KNN (**A**) and the 3-months KNN–KNN (**B**) and they are reported on the x-axis, after ordering features by the mean of the absolute SHAP value. Most relevant marginal interactions are represented for both the admission model (**C**,**D**) and the 3-months model (**E**).

The calculation of SHAP values from the best admission KNN resulted in age being the feature with the strongest weight in predicting GOS-E > 4. Older age, presence of more invasive feeding strategies, higher disability rates (lower DRS) were all found affecting the prediction of GOS-E (Fig. 3A). When evaluating marginal joint probabilities between predictors, a clear interaction effect resulted between age and CRS-R communication score (Fig. 3C). Specifically, beside the inverse relationship between age and its SHAP value ($$R = -0.948$$), an increase of the effect of the age on the GOS-E is seen in patients with higher communication levels. In particular, older age has a significatively more negative effect on the GOS-E in patients with higher CRS-R communication values ($$R = -0.981$$ in patients with $$CRS-{R}_{comm}=1$$ vs $$R = -0.950$$ in patients with $$CRS-{R}_{comm}>1)$$. Also, the age-VS interaction significantly influenced the value of the age SHAP value ($${p}_{interaction}\left[age \times VS\right]<0.001$$, OR 1.165, CI 1.137–1.192) similarly to CRS-R communication score.

Furthermore, the interaction effect between the admission state of consciousness and the type of feeding showed how enteral nutrition contributes negatively to the GOS-E prediction when patient are mostly in MCS (${p}_{interaction}\left[PEG \times VS\right]<0.001$, OR 0.603, CI 0.526–0.690). The negative effect of the presence PEG, almost turns neutral in patients in a VS (Fig. 3D). Conversely, the ‘positive’ effect of being fed via NGT results significantly stronger in patients in a VS than in a MCS (${p}_{interaction}[NGT \times VS]<0.001$, OR 0.826, CI 0.720–0.948).

The highest absolute SHAP values in the KNN–KNN solution were observed for the normalized GOS-E prediction of the admission KNN, followed by MC_tot_ (Fig. 3B). Coherently, higher GOS-E predictions at admission yielded positive contributions on the 3-months GOS-E prediction as well as a lower number of MCs. The interaction between the combined effects of the GOS-E admission prediction and MC_tot_ (Fig. 3E) showed that the KNN-KNN model assigned a stronger importance to the GOS-E prediction at admission in patients with fewer MCs (${p}_{interaction}\left[GOS-{E}_{T0}\times M{C}_{tot}\right]<0.001$, OR 1.355, CI 1.280–1.433). Overall, a negative absolute contribution to the GOS-E prediction was also provided by a higher number of respiratory and musculo-skeletal complications.

## Discussions

An accurate prognosis of pDoC is crucial for optimizing patients’ management, but clinicians often have to deal with a high variability in patients’ clinical conditions throughout the rehabilitative path, in particular due to frequent occurrence of medical complications. In this multicenter, longitudinal study we enrolled a cohort of 176 patients with prolonged VS/UWS and MCS and evaluated their functional outcome up to 6 months after traumatic or non-traumatic brain injury. Because of the importance of a dynamically-evolving prognostication^33^, we developed a cascade ML model that, beside training and validating a model at admission, we added data collected at 3 months onto the prediction of the admission model. Differently from previous studies in literature^16,32^, in the present work data at admission and at 3 months were used in two separate models, connected by the prediction of the first model. In this way, it is possible to investigate the effect of MCs (risen in the first 3 months of stay in rehabilitative units) on the prediction done at admission. Furthermore, it does not only lead to the evaluation of accuracy changes between admission and 3-months based models, but also it relates the severity/number of medical complications developed with the prediction done at admission. Such techniques, based on the evaluation of marginal probabilities after calculation of Shapley values allow to investigate the interaction effects between independent variables, without increasing the cardinality of the predictors. With such methods, data-driven confirmations of known trends in the prognosis of patients with a DoC could be retrieved.

We considered as an outcome of interest the level of disability as classified by the GOS-E score. Notwithstanding even a minimal improvement in the level of consciousness can be important for patients’ relatives^50^ and for identifying patients with higher likelihood of further clinical improvement^51^, here we aimed at predicting the level of functional independence according to a 30-year literature^52,53^.

Our ML algorithm, based only on demographical and clinical data collected at admission, was able to predict patients’ functional outcome at 6 months with 88.6% accuracy. We observed that younger age, higher CRS-R total and sub-scores, absence of an enteral feeding device and lower DRS total score were significant predictors of a favorable outcome. Several studies demonstrated that age is one of the main predictors of the functional improvement in patients with pDoC^15,32,54^, probably in relation with more premorbid medical illnesses^55^ and lower age-related brain plasticity^56^ in the elderly. In addition, we found a significant interaction between age and the level of consciousness at admission. More precisely, results showed that the weight of age in predicting the functional outcome increased as a function of the entry diagnosis (i.e., MCS rather than VS/UWS) and of the CRS-R communication sub-score. It could be speculated that the influence of age might be negligible in VS/UWS patients, that are often characterized by a more severe brain injury^57,58^ and worse general clinical conditions^54^ with respect to patients in MCS. A similar explanation might be applied to the interaction between age and CRS-R communication sub-score since the communication sub-scale of the CRS-R collected at admission could only assume the 0 or 1 values^59^, indicating respectively a diagnosis of VS/UWS or MCS, and actually representing a dichotomic index of level of consciousness.

We found a significant role of the CRS-R total score in predicting a favorable outcome. This finding supported the prognostic validity of CRS-R total score, in keeping with previous longitudinal studies on clinical improvement^6,13,54,60,61^ and disability level^61^ in individuals with pDoC. Since the CRS-R total score summarizes the scores of six sub-scales with hierarchically-organized items, it could be considered an indirect index of severity of brain injury^62^, with higher scores corresponding to higher-level neurologic functioning.

Similarly, we observed that the level of disability at admission as assessed by the DRS was a significant predictor of the GOS-E score at 6 months. This result is in line with previous literature^5,54,63^ suggesting a strict relationship between these two clinical tools evaluating functional independence.

We also found that the lack of enteral feeding device is associated with favorable functional outcome, and that this effect is more evident for patients in MCS than in VS/UWS. This finding would confirm that early recovery of non-automatic oral feeding is related to recovery of higher cognitive function, particularly in patients in MCS, in whom conscious behavioral responses can be present^64^.

Our model was improved by including information on patients’ MCs collected within the first 3 months of intensive rehabilitation, resulting in a prediction accuracy of 92.6%. In particular, the cascade model showed how the update of the predictions directly reflects the medical complications risen in the first 3 months of stay in rehabilitation units, by correcting the GOS-E admission prediction proportionally with MC_tot_. In other words, for patients who had fewer MCs in the first 3 months, the GOS-E predicted at admission had a higher impact than for patients who had more MCs. This finding is in line with previous studies showing that MCs arising in the first 3 months may significantly affect functional outcomes^15,17,20–24^.

Our results are in line with the study of Lucca et al. ^32^, which reported an area under the curve of 0.876 when predicting changes in functional disabilities (assessed by DRS) by using data taken during intensive care and at admission to rehabilitation unit. In that study, the impact of MCs on the prediction of functional outcomes was evaluated via a multivariate regression analysis which allows for the investigation of independent predictors but cannot cope with interaction effects. By these means, the authors found that the total number of MCs was significantly associated with the worse outcome. Coherently, our univariate findings showed that a smaller number of MCs increased likelihood of better recovery. Moreover, we also found the presence of respiratory complications to be predictive of a worse functional outcome, as in previous works targeting both level of consciousness^36^ and functional independence^22,24^. Moreover, the authors reached similar results targeting the dichotomized version of the GOS-E scale and reporting a validation area under the curve of 0.78 with models trained with data taken at admission^63^. It must be mentioned, though, that our case mix included TBIs, anoxic and vascular etiologies notably increasing the complexity of the prediction task with respect to only TBIs, as in Farzaneh et al.^65^.

In this respect, our analysis comparing traumatic versus vascular versus anoxic etiology did not reveal significant association with outcome. Even contrasting traumatic versus non-traumatic etiology (i.e., grouping vascular and anoxic etiology in one non-traumatic subsample) we did not find significant association with outcome (p = 0.575, χ^2^ = 0.315). The lack of association of etiology with outcome in our sample could seem at odds with previous studies reporting a better prognosis in individuals with traumatic brain injury^63^. However, it must be considered that the studies on the relationships between traumatic brain injury and outcome have often been performed at early stages of disease, whereas here we dealt with post-acute rehabilitative stage and focused on survivors who had not recovered consciousness within 1 month post onset. At this stage, information about etiology seems not able to provide solid prognostic cues for recovery. This consideration is consistent with findings from several studies on samples with similar features, i.e. with prolonged DoC^6,54^.

Analogously, we must also comment on the fact that at admission other possible predictors of outcome, such as tracheostomy and non-invasive ventilatory support did not significantly differ between patients with good or poor outcome. These findings could be likely ascribed to the fact that these factors were present in most individuals enrolled in this study, and so could hardly discriminate individuals with different outcomes. For instance, tracheostomy was present in the 95.3% of patients with GOS-E < 4 at discharge and 85.7% of patients with GOS-E > 4, and the difference in frequency of tracheostomy between the two groups only approached the significance level (p = 0.055). By the same reasoning, ventilatory support was very frequent. In particular, ~ 50% of patients in respectively the favorable/unfavorable outcome received a respiratory support (O_2_ support or non-invasive ventilation). Also, merging persons with O_2_ support and persons with non-invasive ventilatory support and comparing them with those without any support, we did not find significant associations with outcome (p = 0.373, χ^2^ = 0.792). Also, the median value for musculo-skeletal-cutaneous complications was found to be 1.5 in the GOS-E < 4 and of 1 in the GOS-E > 4 group, not allowing for a distinction between the two groups.

The present study had several limitations. Firstly, it must be acknowledged that clinical characteristics related to the ICU period can notably affect the outcome. Therefore, by adding such characteristics (e.g., Marshall scores, ICU vital supports) model accuracy could certainly be improved, but these data were not available. Furthermore, interactions between acute setting features, admission variables and MCs risen within the first 3 months would be allowed to emerge. Secondly, pharmacological interventions (beta-blockers, etc.) may affect/prevent the emergence of some symptoms (e.g. paroxysmal sympathetic hyperactivity), but could not be included in analysis since out of the scope of this work. Thirdly, the generalizability of the proposed model needs to be tested on prospective external validation sets and the improvements carried into clinical practice must be evaluated following standard decision support tools validation procedures. Nevertheless, the multicentric nature of the study (N = 23), the geographically distant hospitals and the validation approach implemented ensures the validity of the results. Overall, the proposed algorithm works with no instrumented data, avoiding costly and invasive examinations (e.g. fMRI^66^) and utilizing straightforward checklists and clinical scales. Interpretable and explainable models as the one proposed here, not only could increase trustworthiness of the solution, but also provide contributions of the features to the predictions in a patient-specific manner. Fourthly, a limitation in the dichotomization of the GOS-E score might result in the ceiling effect on the range of good recovery^67^. In our case mix (only patients with a DoC) only 7 (4%) and 3 (1.7%) patients respectively scored 7 and 8 on the GOS-E scale 6 months after the event. This finding shows how such ceiling effect is negligible when evaluating patients with acquired prolonged DoC with respect to patients with only a sABI who recovered consciousness at an earlier stage after onset.

A last limitation could relate to the type of coding we used for medical complications. Our checklist coded medical complications as a function of the system/organ involved, and did not distinguish them for type or etiology (e.g., as far as the genito-urinary tract is concerned, infections, bleeding, urinary stones, urinary obstructions and renal insufficiency were merged in the same category). For this reason, we could have underestimated the prognostic value of infectious conditions, such as those of the urinary tract or of the respiratory system which are very frequent in individuals with prolonged DoC, although, by considering them within medical complications of the respective system/organ, we took into account their possible contribution to outcome prediction. By the same token, we considered spasticity (one possible predictor of outcome in DoC^68^) among the pathologies of musculoskeletal-cutaneous system, so we could not evaluate the individual prognostic value of spasticity alone. Future studies might obtain further prognostic information by adopting more fine-grained checklist for medical complications.

In conclusion, ML offers promising and automated medical reporting and prognosis algorithms, but at the moment such models are rarely deployed in daily clinical settings^65^. To improve transparency and practicality, we proposed a machine learning-based framework that is explainable and that is based on affordable features, with no instrumental requirements. Using this model, we found that functional outcome of patients with pDoC at 6-month post-injury can be predicted at admission with an accuracy of 88.6%. Thereafter, the adjustment of this model with information on MCs arising within the first 3 months of hospitalization led the accuracy of prediction to 92.6%. Such accuracies were obtained with relatively simple algorithms, increasing the generalization capabilities of the solution, demonstrating how even classical ML techniques may be sufficient to accurately predict functional outcome of patients with DoC. Surely, with more complex/deep models better accuracies may be obtained although conditioned to the need of having a much greater number of samples. Overall, we believe that this model could effectively support clinicians and patients’ caregivers in the decision-making about treatment and rehabilitative path.

## Supplementary Information

Supplementary Information.

## Data Availability

The data that support the findings of this study will be made available from the corresponding author upon request for replication purposes.
