# Supplementary material for: Predicting outcome of patients with prolonged disorders of consciousness using machine learning models based on medical complexity
Source: Sci Rep. 2022 Aug 5;12:13471. doi: 10.1038/s41598-022-17561-w (PMC9356130; doi:10.1038/s41598-022-17561-w)
Supplement: Supplementary file 1 — Supplementary Information. [file 41598_2022_17561_MOESM1_ESM.docx]

Supplementary material A

Checklist for categorization of medical complication

| **Category** | **Description** |
| --- | --- |
| Endocrine-metabolic | Metabolic abnormalities (e.g. hyponatremia, anemia, hypoalbuminemia) and endocrine disorders (e.g.diabetes mellitus, thyroid gland dysfunctions) |
| Cardio-Vascular | Heart failure (e.g. acute myocardial infarction, congestive heart failure) and/or acute arrhythmia (e.g. atrial fibrillation, ventricular tachycardia), and/or arterial-vein thrombosis |
| Musculo-skeletal-cutaneous | Hypertonia/spasticity, fractures, arthritis, pressure sores |
| Gastrointestinal | Bleeding, bowel obstruction, peritonitis, Clostridium difficile enteritis, diarrhea, biliary lithiasis, hepatitis, pancreatitis |
| Genito-urinary tract | Infections, bleeding, urinary stones, urinary obstructions, renal insufficiency |
| Respiratory | Pulmonary infections, tracheal stenosis or malacia, tracheoesophageal fistula, central respiratory drive deficits |
| Neurological/Neurosurgical | Hydrocephalus, new brain injury, ventriculoperitoneal shunt dysfunction, cranioplasty infection. |
| Epilepsy/Myoclonus | Partial or generalized seizures, spontaneous or reflex myoclonus |
| Heterotopic Ossification | Range of motion limitation and/or articular pain and/or local inflammatory signs in joint due to radiologically evident abnormal mature lamellar bone in extra-skeletal soft tissues |
| Paroxysmal sympathetic hyperactivity | Paroxysmal episodes characterized by increased heart rate, respiratory rate, diaphoresis, motor hyperactivity with or without increased blood pressure and/or hyperthermia |

Supplementary material B

GOS-E scale categories and rating

| **GOSE-E value** | **Category** |
| --- | --- |
| 1 | Death |
| 2 | Vegetative State |
| 3 | Lower severe disability |
| 4 | Upper severe disability |
| 5 | Lower moderate disability |
| 6 | Upper moderate disability |
| 7 | Lower good recovery |
| 8 | Upper good recovery |

Supplementary material C

The list of local ethics committees/institutional review boards (IRB):

1. Fondazione Pascale IRCCS, Napoli for: 1. Dept. of Acquired Brain Injury, Fondazione Don Carlo Gnocchi, Sant'Angelo dei Lombardi, Italy and 2. Lab for DoC Study, Istituti Clinici Scientifici Maugeri IRCCS, SB S.p.A. Lab for DoC Study, Telese Terme, Italy.

2. Department of Acquired Brain Injury, IRCCS Fondazione Don Carlo Gnocchi, Florence, Italy.

3. Neurorehabilitation Unit, HABILITA Zingonia/Ciserano, Bergamo, Italy.

4. Department of Biotechnological and Applied Clinical Sciences, Neurological Institute, University of L'Aquila, L'Aquila, Italy.

5. Unità di Neuroriabilitazione Cognitiva, Istituti Clinici Zucchi, Carate Brianza, Italy.

6. IRCCS Centro Neurolesi "Bonino-Pulejo", Messina, Italy.

7. Unità di Riabilitazione Gravi Cerebrolesioni, Istituto S. Anna, Crotone, Italy.

8. Unità Medicina Riabilitativa Neuromotoria, Istituti Clinici Scientifici Maugeri IRCCS, SB S.p.A, Pavia, Italy.

9. Casa dei Risvegli Luca De Nigris, IRCCS Ospedale Maggiore, Bologna, Italy.

10. Dip. di Riabilitazione, F.T. Camplani Clinica Ancelle Carità, Cremona, Italy.

11. Unità Post-Coma e di Ricerca Traslazionale, IRCCS, Fondazione Santa Lucia, Rome, Italy.

12. U.F. Riabilitazione Neuromotoria, Istituto Clinico Quarenghi, S. Pellegrino Terme, Italy.

13. Dip. di Neurologia - UGCA, Ospedale S. Giovanni Battista, Foligno, Italy.

14. Centro Cardinal Ferrari, S. Stefano Riabilitazione, Fontanellato di Parma, Italy.

15. Severe Acquired Brain Injuries Dept Section, Integrated Care Dept of Medical Specialties, Azienda Ospedaliero-Universitaria Pisana, Pisa, Italy.

16. Centro Medicina del Sonno, Ospedale Sacro Cuore Don Calabria, Verona, Italy.

17. Unit for Severe Acquired Brain Injuries, Rehabilitation Dept, Giuseppe Giglio Foundation, Cefalù, Italy.

18. Dip. Neuroscienze e Riabilitazione, Azienda Ospedaliera Brotzu, Cagliari, Italy.

19. Unità Medicina Fisica e Riabilitazione, Ospedale Riabilitativo Di Marzana, Verona, Italy.

20. Unità di Medicina Fisica e Neuroriabilitazione, IRCCS "Casa Sollievo della Sofferenza", San Giovanni Rotondo, Italy.

21. Unità di Medicina Riabilitativa Intensiva, Ospedale Castel San Giovanni, Italy.

22. Struttura di Riabilitazione Neuromotoria, Presidio Ospedaliero Vimercate, Monza, Italy.

23. Presidio di Riabilitazione Neuromotoria, Azienda Socio Sanitaria Territoriale dei Sette Laghi, Cuasso Al Monte, Italy.

Supplementary material D

Hyperparameters optimization results for all EN models (appendix **D1**), OMP models (appendix **D2**) and KNN models (appendix **D3**). The first row of each table corresponds to the admission model whilst from second to fourth row, the 3-months model are reported.

| **C1** | C | L1_ratio_ | OV_rate_ | UN_rate_ |
| --- | --- | --- | --- | --- |
| EN | 0.3 | 0.54 | 4 | 6 |
| EN-EN | 0.1 | 0.66 | 3 | 7 |
| OMP-EN | 0.24 | 0.46 | 3 | 6 |
| KNN-EN | 0.21 | 0.54 | 4 | 8 |

| **C2** | $N_{coeff\neq0}$ | OV_rate_ | UN_rate_ |
| --- | --- | --- | --- |
| OMP | 2 | 8 | 4 |
| EN-OMP | 5 | 6 | 3 |
| OMP-OMP | 4 | 8 | 5 |
| KNN-OMP | 2 | 4 | 7 |

| **C3** | $N_{\mathrm{neighbors}}$ | OV_rate_ | UN_rate_ |
| --- | --- | --- | --- |
| KNN | 6 | 10 | 3 |
| EN-KNN | 2 | 7 | 8 |
| OMP-KNN | 5 | 4 | 8 |
| KNN-KNN | 2 | 7 | 7 |
